# Corneal Curvature Radius and Associated Factors in Chinese Children: The Shandong Children Eye Study

**DOI:** 10.1371/journal.pone.0117481

**Published:** 2015-02-06

**Authors:** Yue Ying Zhang, Wen Jun Jiang, Zhao E. Teng, Jian Feng Wu, Yuan Yuan Hu, Tai Liang Lu, Hui Wu, Wei Sun, Xing Rong Wang, Hong Sheng Bi, Jost B. Jonas

**Affiliations:** 1 The First College of Clinical Medicine, Shandong University of Traditional Chinese Medicine, Jinan, Shandong, China; 2 Institute of Basic Medicine, Shandong Academy of medical sciences, Jinan, Shandong, China; 3 Eye Institute of Shandong University of Traditional Chinese Medicine, Jinan, Shandong, China; 4 Department of Ophthalmology, Wulian People’s Hospital, Rizhao, Shandong, China; 5 Department of Ophthalmology, Shandong University of Traditional Chinese Medicine, Jinan, Shandong, China; 6 Department of Ophthalmology, The Second Affiliated Hospital of Shandong University of Traditional Chinese Medicine, Jinan Shandong, China; 7 Department of Ophthalmology, Medical Faculty Mannheim of the Ruprecht-Karls-University Heidelberg, Germany; Dalhousie University, CANADA

## Abstract

**Purpose:**

To investigate the distribution of the (CCR) and its associated factors in children.

**Methods:**

Using a random cluster sampling method, the school-based, cross-sectional Shandong Children Eye Study included children aged 4 to 18 years from the rural county of Guanxian and the city of Weihai in the province of Shandong in East China. CCR was measured by ocular biometry.

**Results:**

CCR measurements were available for 5913 (92.9%) out of 6364 eligible children. Mean age was 10.0±3.3 years, and mean CCR was 7.84±0.27 mm (range: 6.98 to 9.35 mm). In multivariate linear regression analysis, longer CCR (i.e. flatter cornea) was significantly associated with the systemic parameters of male sex (P<0001;standardized regression coefficient beta: -0.08;regression coefficient B:-0.04; 95% Confidence Interval (CI):-0.05,-0.03), younger age (P<0.001;beta:-0.37;B:-0.03;95%CI:-0.04,-0.03), taller body height (P = 0.002;beta:0.06;B:0.001;95%CI:0.000,0.001), lower level of education of the father (P = 0.001;beta:-0.04;B:-0.01;95%CI:-0.02,-0.01) and maternal myopia (P<0.001;beta:-0.07;B:-0.04;95%CI:-0.06,-0.03), and with the ocular parameters of longer ocular axial length (P<0.001;beta:0.59;B:0.13;95%CI:0.12,0.14), larger horizontal corneal diameter (P<0.001;beta:0.19;B:0.13;95%CI:0.11,0.14), and smaller amount of cylindrical refractive error (P = 0.001;beta:-0.09;B:-0.05;95%CI:-0.06,-0.04).

**Conclusions:**

Longer CCR (i.e., flatter corneas) (mean:7.84±0.27mm) was correlated with male sex, younger age, taller body height, lower paternal educational level, maternal myopia, longer axial length, larger corneas (i.e., longer horizontal corneal diameter), and smaller amount of cylindrical refractive error. These findings may be of interest for elucidation of the process of emmetropization and myopization and for corneal refractive surgery.

## Introduction

The anterior corneal curvature radius (CCR) determines two third of the optical refractive power of the eye and is thus an important parameter of the ocular optical system. Abnormal values of the CCR, such as keratoconus or cornea plana, lead to severe refractive ametropias and any disparity in CCR between the corneal meridians is the cause of corneal astigmatism. In particular in infants and young children, any abnormal CCR can be the cause of amblyopia. Numerous previous population-based studies have examined the normative values and distribution of CCR, the prevalence of diseases characterized by abnormal CCRs such as keratoconus, and factors associated with CCR in adult populations of various ethnicities [1–9]. These studies, however, were focused on adults with an age of ≥40 years, while relatively few investigations were conducted to evaluate distribution and associated factors of CCR in children [10–17]. The latter studies often included only children of special age groups, such as an age group of 7 to 9 years or an age group of less than six months or they were not population-based. In studies on a limited age spectrum however, a multivariate analysis could not fully explore the role of factors such as general anthropomorphic measurements (e.g. body height, age) and socioeconomic data which may potentially be associated with CCR. Consequently, while these studies showed that higher CCR was associated with older age and taller body height in neonates and infants aged less than one year, it has remained unclear whether CCR is associated with age and body height in children with an age of more than four years. While it was reported that CCR was positively associated with older age and taller body height in Singapore Chinese children with an age of 7 to 9 years, the Aston Eye Study demonstrated that children in Birmingham / UK did not differ in CCR between the age of 6 to 7 years and the age of 12 to 13 years [13, 18]. We therefore conducted this school-based study on children during the whole age spectrum of adolescence from 4 to 18 years, measured the CCR, assessed other ocular and general parameters, evaluated the distribution of CCR, and examined potential associations between CCR and other ocular factors and systemic parameters in multivariate analysis. We performed this study in the Eastern Chinese province of Shandong, since China is the country which may most be affected by the marked increase in the prevalence of myopia in particular in the young generations, in a manner, that already nowadays myopia has been feared as one of the upcoming most common causes for visual impairment and blindness in the next older generations of China [19].

## Method

The Shandong Children Eye Study was a school-based cross-sectional study in East China. The Ethics Board of the Eye Institute of the Shandong University of Traditional Chinese Medicine and the local Administration of the Education and School Board approved the study, and the parents or guardians of the children gave written consent, according to the Declaration of Helsinki. The study was performed in the city of Weihai in the eastern part of Shandong, and in the rural areas of Guanxian in the west of Shandong. Kindergarten, elementary schools, junior high schools and senior high schools in Guanxian and in Weihai were randomly selected or the study. The age of the participants ranged between four years to 18 years. The study has been described in detail recently [20,21].

All study participants underwent an interview with standardized questions. The questionnaire consisted of questions on the children’s family history, time spent outdoors and the activities performed outdoors, time spent indoor and the activities carried out indoors, intensity of learning and studying for school, lifestyle, etc. The structure of the questionnaire was similar to the design of the questionnaire used previously in the Refractive Error Study in Children (RESC) studies [22]. General and ophthalmic examinations were then carried out. Performing auto-refractometry (KR-8900, Topcon, Itabashi, Tokyo, Japan), we measured the spherical refractive error and cylindrical refractive error, corneal astigmatism, and CCR. If the difference between the maximum and minimum value of the spherical refractive error or cylindrical refractive error were less than 0.5 diopters, the measurement were repeated. The spherical equivalent of refractive error was defined as the spherical value of refractive error plus one half of the cylindrical value. All refractive errors were examined under cycloplegia, which was achieved by applying 1% cyclopentolate eye drops (Alcon, Ft. Worth, Texas, USA) at least thrice. Intraocular pressure was measured by a non-contact tonometer (CT-80A, Topcon, Co., Tokyo, Japan). Ocular biometric parameters such as axial length and corneal horizontal diameter were measured by biometry (IOL- Master; V5.0, Carl Zeiss Meditec AG, Jena, Germany). The axial length / CCR ratio (AL/CCR) was calculated by dividing the axial length value by the CCR. All examinations were undertaken by trained ophthalmologists and optometrists.

Statistical analysis was performed using a commercially available statistical software package (SPSS for Windows, version 21.0, IBM-SPSS, Chicago, IL, USA). In a first step, we determined the mean value (presented as mean ± standard deviation) of the main outcome parameters. In a second step, we performed univariate analyses of the associations between CCR and other ocular and systemic parameters. In a third step, a multivariate analysis was performed with CCR as dependent parameter and all of those parameters as independent parameters which were significantly associated with CCR in the univariate analyses. We first removed parameters for which the analysis showed a collinearity. We then removed all those parameters that were no longer significantly associated with CCR in the multivariate analysis in a step-by-step manner, through a combination of stepwise, forward, and backward regression analysis. In case of doubt, we kept those parameters that were considered to be closer related to CCR and dropped those parameters which were secondarily associated with CCR. Odds ratios (OR) and 95% confidence intervals (95%CI) were calculated. All *P*-values were 2-sided and were considered statistically significant when the values were less than 0.01.

## Results

Out of 6364 eligible children fulfilling the inclusion criterion of an age from 4 to 18 years, CCR measurements were eventually available for 5913 (92.9%) participants (2792 (47.2%) girls). The examination was refused by 328 (5.2%) children and 123(1.9%) children did not accomplish the whole general and ophthalmic examination to gain reliable CCR measurements. The mean age was 10.0 ± 3.3 years (median: 10.0 years; range, 4 to 18 years), and the mean refractive error (spherical equivalent) was −0.21 ± 2.10 diopters (median: 0.50 diopters; range: −11.75 to 10.5 diopters) for the right eyes and-0.13 ± 2.08 diopters (median: 0.50 diopters; range: -11.75 to 11.00 diopters) for the left eyes.

**Fig 1:**
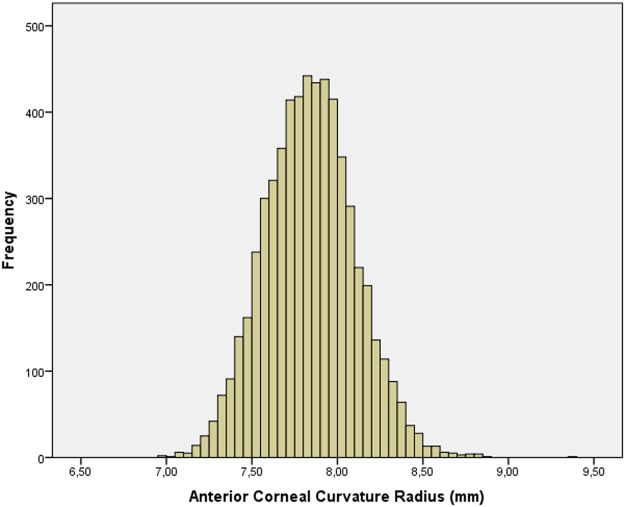
Histogram Showing the Distribution of the Anterior Corneal Curvature Radius in the Shandong Children Eye Study.

The mean CCR was 7.84 ± 0.27 mm (median: 7.84 mm; range: 6.98 to 9.35 mm) (Table 1; Fig. 1) for right eyes. Mean CCR of the left eyes was 7.84 ± 0.26 mm (median: 7.84 mm; range: 6.98 to 9.36 mm) (Table 1). The measurements of CCR were significantly (*P*<0.001) higher in the left eyes than in the right eyes. The mean absolute amount of the inter-eye difference was 0.050 ± 0.059 mm. CCR was not normally distributed for both eyes (Kolmogorov-Smirnov test, *P* = 0.001, and *P*<0.001, respectively).

**Table 1:** Mean Corneal Curvature Radius (Mean ± SD) in the Shandong Children Eye Study Stratified by Age, Sex and Region of Habitation.

Age (Years)	n	Corneal Curvature Radius (mm), Right Eyes	Corneal Curvature Radius (mm), Left Eyes	Mean Inter-Eye Difference in Corneal Curvature Radius (mm), Absolute Amount
4	113	7.818 ± 0.279	7.829 ± 0.276	0.047 ± 0.004
5	354	7.843 ± 0.263	7.849 ± 0.261	0.044 ± 0.002
6	438	7.835 ± 0.256	7.841 ± 0.250	0.068 ± 0.003
7	637	7.832 ± 0.272	7.842 ± 0.278	0.067 ± 0.003
8	740	7.821 ± 0.256	7.823 ± 0.259	0.068 ± 0.003
9	543	7.834 ± 0.259	7.838 ± 0.256	0.080 ± 0.003
10	700	7.860 ± 0.263	7.865 ± 0.264	0.052 ± 0.002
11	557	7.839 ± 0.261	7.842 ± 0.259	0.067 ± 0.003
12	462	7.843 ± 0.257	7.845 ± 0.257	0.038 ± 0.002
13	432	7.851 ± 0.281	7.860 ± 0.275	0.050 ± 0.002
14	338	7.852 ± 0.287	7.856 ± 0.285	0.041 ± 0.002
15	230	7.831 ± 0.255	7.837 ± 0.254	0.038 ± 0.003
16	134	7.818 ± 0.288	7.818 ± 0.272	0.054 ± 0.005
17	128	7.776 ± 0.250	7.782 ± 0.255	0.044 ± 0.004
18	107	7.793 ± 0.263	7.799 ± 0.266	0.038 ± 0.004
Sex				
Boys	3121	7.898 ± 0.260	7.903 ± 0.260	0.050 ± 0.066
Girls	2792	7.768 ± 0.253	7.773 ± 0.251	0.049 ± 0.050
Region of Habitation				
Rural	3016	7.806 ± 0.260	7.812 ± 0.256	0.051 ± 0.064
Urban	2897	7.868 ± 0.266	7.873 ± 0.269	0.049 ± 0.054
Total	5913	7.836 ± 0.265	7.842 ± 0.264	0.050 ± 0.059

**Fig 2:**
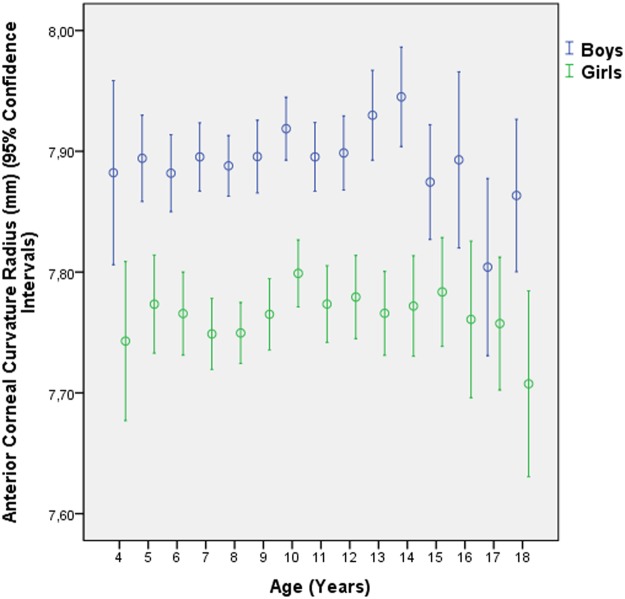
Distribution of the Anterior Corneal Curvature Radius Stratified by Age and Sex in the Shandong Children Eye Study.

In univariate analysis, CCR was not significantly (*P* = 0.08) associated with age within the age period from four to 14 years. Beyond an age of 14 eyes, CCR was significantly (*P* = 0.01) lower than for the age period of 14 or less years (Fig. 2). If the study population was stratified by sex, CCR was significantly lower in the older group than in the younger group for boys (*P* = 0.03, while the difference was not significant for girls (*P* = 0.55) (Fig. 2).

**Fig 3:**
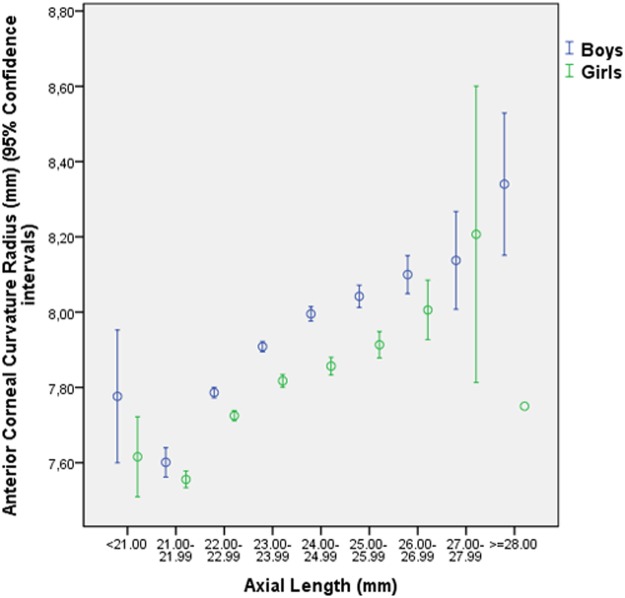
Diagram Showing the Distribution of the Anterior Corneal Curvature Radius Stratified by Axial Length and Sex in the Shandong Children Eye Study.

In univariate analysis, higher CCR was significantly associated with the systemic parameters of male sex (*P*<0.001), higher school grade (*P* = 0.01), urban region of habitation (*P*<0.001), taller body height (*P*<0.001), heavier body weight (*P*<0.001), higher body mass index (*P*<0.001), heavier birth weight (*P* = 0.01), higher paternal and maternal level of education (*P*<0.001), less maternal myopia (*P* = 0.007), more total time spent outdoors (*P* = 0.001), more time spent using a computer (*P*<0.001), brighter illumination during studying (*P*<0.001), more intake of rice (*P* = 0.002) and red meat (*P*<0.001) and protein-rich food (*P*<0.001), less intake of wheat associated food (*P*<0.001), less intake of sweet food (*P* = 0.02) (Table 2). Higher CCR was significantly associated with the ocular parameters of higher (i.e., hyperopic) refractive error (*P* = 0.03), higher cylindrical refractive error (*P*<0.001), longer axial length (*P*<0.001) (Fig. 3), longer horizontal corneal diameter (*P*<0.001), and higher intraocular pressure measurements (*P*<0.001) (Table 2). CCR was not significantly associated with paternal myopia (*P* = 0.13), the total time spent indoors (*P* = 0.09), time spent indoors reading or writing (*P* = 0.28), and the time spent indoors watching television or movies (*P* = 0.52).

**Table 2:** Univariate Analysis of Associations between Corneal Curvature Radius (mm) (Right Eyes) and Non Ocular and Ocular Parameters in the Shandong Children Eye Study.

Parameter	*P* Value	Standardized Correlation Coefficient	Steepness of the Regression Line	95% CI of the steepness of Regression Line
Non Ocular Parameter				
Boys / Girls	<0.001	-0.246	-0.130	-0.144, -0.117
School Grade	0.014	0.033	0.003	0.001, 0.005
Region of Habitation (Rural / Urban)	<0.001	0.118	0.063	0.049, 0.076
Body Height (cm)	<0.001	0.082	0.001	0.001, 0.001
Body Weight (kg)	<0.001	0.102	0.002	0.001, 0.002
Body Mass Index (kg/m^2^)	<0.001	0.099	0.007	0.005, 0.009
Birth Weight (kg)	0.014	0.046	0.003	0.001, 0.005
Paternal Educational Level	<0.001	0.063	0.014	0.008, 0.021
Maternal Education Level	<0.001	0.072	0.017	0.010, 0.023
Maternal Myopia	0.007	-0.04	-0.03	-0.04, -0.01
Time Spent Outdoors (Total) (Hours)	0.001	0.049	0.004	0.002, 0.006
Time Spent Using Computer (Hours)	<0.001	0.014	0.066	0.008, 0.020
Illumination during Studying	<0.001	0.051	0.016	0.008,0.025
Intake of Wheat Associated Food	<0.001	-0.051	-0.014	-0.022, -0.007
Intake of Rice	0.002	0.043	0.012	0.004, 0.019
Intake of Sweet Food	0.013	-0.035	-0.01	-0.017, -0.002
Intake of Red Meat (Including Beef, Pork, Mutton and Liver of Animals)	<0.001	0.058	0.017	0.009, 0.025
Intake of Protein-Rich Food (Including Milk, Egg, Bean, Meat)	<0.001	0.070	0.007	0.004, 0.010
Ocular Parameters				
Refractive Error (Spherical Equivalent) (Diopters)	0.025	0.029	0.004	0.000, 0.007
Cylindrical Refractive Error (Diopters)	<0.001	0.145	0.065	0.053, 0.076
Axial Length (mm)	<0.001	0.343	0.058	0.054, 0.062
Horizontal Corneal Diameter (mm)	<0.001	0.232	0.105	0.094, 0.116
Intraocular Pressure Measurements (mmHg)	0.007	0.035	0.003	0.001, 0.006

In the multivariate linear regression analysis, we dropped the parameter of body weight due to collinearity (VIF: 58) with body height and body mass index, the parameter of school grade (VIF: 5.09), and the parameter of refractive error due to its collinearity with axial length. We then dropped in a step-wise manner those parameters which were no longer significantly associated with CCR in the multivariate model: birth weight (*P* = 0.49), intake of red meat-rich food (*P* = 0.91), intake of sweet food (*P* = 0.75), level of maternal education (*P* = 0.88), food rich in wheat product (*P* = 0.66), time spent using a computer (*P* = 0.90), body mass index (*P* = 0.92), illumination during reading (*P* = 1.00), food rich in rice (*P* = 0.86), intraocular pressure (*P* = 0.61), total time spent outdoors (*P* = 0.54), intake of protein-rich food (*P* = 0.80), and region of habitation (*P* = 0.09). In the final model, longer CCR (i.e. flatter cornea) was significantly associated with the systemic parameters of male sex (*P*<0001), younger age (*P*<0.001), taller body height (*P* = 0.002), lower level of education of the father (*P* = 0.001) and maternal myopia (*P*<0.001), and with the ocular parameters of longer ocular axial length (*P*<0.001), larger horizontal corneal diameter (*P*<0.001), and smaller amount of cylindrical refractive error (*P* = 0.001) (Table 3).

**Table 3:** Multivariate Linear Regression Analysis of Associations Between Corneal Curvature Radius (mm) (Right Eye), and Non Ocular and Ocular Parameters in the Shandong Children Eye Study.

Parameter	*P*-Value	StandardizedRegression Coefficient Beta	Regression Coefficient B	95% Confidence Interval of B	Variance Inflation Factor
Age (Years)	<0.001	-0.37	-0.03	-0.04, -0.03	3.48
Boys / Girls	<0.001	-0.08	-0.04	-0.05, -0.03	1.10
Body Height (cm)	0.002	0.06	0.001	0.000, 0.001	3.28
Maternal Myopia	<0.001	-0.07	-0.04	-0.06, -0.03	1.16
Paternal Level of Education	0.001	-0.04	-0.01	-0.02, -0.01	1.17
Axial Length (mm)	<0.001	0.59	0.13	0.12, 0.14	1.84
Horizontal Corneal Diameter (mm)	<0.001	0.19	0.13	0.11, 0.14	1.13
Cylindrical Refractive Error (Expressed in Minus Diopters)	<0.001	0.09	0.05	0.04, 0.06	1.03

## Discussion

In our school-based study in the East Chinese province of Shandong, longer CCR (i.e., flatter corneas) were correlated with female sex, younger age, taller body height, lower paternal educational level, maternal myopia, longer axial length, larger corneas (i.e., longer horizontal corneal diameter), and smaller amount of cylindrical refractive error. Its mean value was 7.84 ± 0.27 mm.

These results partially confirm previous findings and contradict others. In previous study populations, CCR was not significantly associated with age in children [11,12]. In girls, however, CCR showed the tendency of an increase up to the age of 10 years and then a decrease in the age period from 10 to 18 years. In boys, CCR reached a peak at the age of 13 years. A similar tendency was observed in our study in which CCR did not vary significantly up to an age of 14 years after which it decreased significantly (Fig. 2). The Singapore Chinese Children Eye Study showed the CCR increasing with older age in children aged 7 to 9 years [23]. The association between CCR and age may also be of interest for corneal refractive surgery, since they suggest that at least up the age of 18 years, corneal refractive power may not be constant over time.

In agreement with previous investigations such as the Singapore Chinese Children Eye Study and a population-based study of Australian children, CCR was in our study population larger in boys than in girls [15,23]. Similar tendencies were reported for adults [24]. The inter-sex difference in our study (mean CCR in boys: 7.90 ± 0.26 versus mean CCR in girls: 7.77 ± 0.25 mm) of 0.13 mm was similar to the figure reported in previous studies with sex-related differences in CCR ranging between from 0.07 mm to 0.17 mm [25,26].

The mean CCR in the right eye (7.8364 ± 0.2649 mm (median: 7.84 mm; range: 6.98 to 9.36 mm)) was significantly (*P*<0.001) larger than in the left eye (7.8416 ± 0.2642 mm (median: 7.84 mm; range: 6.98 to 9.36 mm)). In view of the relatively small difference between the right eye and left eye, in view of the relatively high number of 5913 study participants, and in view the fact of using a paired sample test which as compared to un-paired sample tests usually shows a higher statistical significance of a difference, one may infer that the statistically significant difference in CCR between the right eye and the left eye may not have a marked clinically importance.

CCR was longer in children with taller body height after adjusting for factors such as sex and age in the multivariate model. It agrees with the findings made in the Avon Longitudinal Study, the Singapore Chinese Children Eye study, and a population-based study of Australian children [13,18,27]. As a corollary, CCR was longer in adults with taller body height, as reported in the Burmese Meiktila Eye Study, the Singapore Malay Eye Study and the Reykjavik Eye Study [5,6,28]. It may reflect a tendency that taller people have larger eyes with larger horizontal and vertical corneal diameters, longer CCR, and longer horizontal and vertical globe diameters [29]. It also agrees with the Children U.K. birth cohort study in which body height and weight growth trajectories, especially shortly after birth, were positively associated with axial length and corneal curvature [30].

In univariate analysis, CCR was associated with heavier birth weight, an association which lost its significance in the multivariate analysis. It may fit with the results of the Avon Longitudinal Study of Parents and Children U.K. birth cohort study in which body height and weight growth trajectories, especially shortly after birth, were positively associated with axial length and corneal curvature [30]. The authors concluded that shared growth mechanisms may contribute to scaling of eye and body size. It also fits with the association between longer CCR (and longer horizontal corneal diameters, i.e. larger corneas) and taller body height as discussed above.

The association between longer CCR and longer axial length are in agreement with previous studies on children and adults [9,31–33]. The correlation suggests that a flatter cornea may (at least partially) compensate for axial elongation to preserve emmetropia in the normal growth of the eye. Interestingly, highly myopic eyes with an axial length of more than 26.5 mm or a myopic refractive error of more than-8 diopters do not show an association between corneal curvature radius and axial length in adults [9].

In the multivariate model, shorter CCR (i.e. steeper corneas) was significantly associated with a higher amount of corneal astigmatism. Similar observations were made in previous studies [34]. Relatively few investigations reported on the association between longer CCR and longer horizontal corneal diameter. In a previous investigation, non-highly myopic eyes with longer CCR had a longer corneal diameter and larger optic disc, suggesting an association between corneal flatness, corneal size and optic nerve head size [34]. It fits with the general notion that large eyes with respect to the horizontal and vertical globe diameters have large optic nerve heads, large corneas, a large retinal surface area and a higher count of retinal photoreceptors, retinal pigment epithelium cells and retinal ganglion cell axons [29,35–37].

Interestingly, CCR was significantly associated with the amount of time spent outdoors or spent indoors with studying in the univariate analysis, while in the final multivariate analysis, these associations were no longer statistically significant. It may suggest that the parameters of time spent indoors and of time spent outdoors have a stronger influence on axial length than on CCR. In multivariate models of previous studies, longer axial length in school children was strongly associated with more time spent indoors or less time spent outdoors [38]. It would explain that in the multivariate analysis of our study, CCR was mainly associated with axial length, while after adjusting for axial length, it was no longer associated with the time spent indoors or the time spent outdoors. It would also fit with anatomical measurements that myopic eyes, and in particular highly myopic eyes, showed a thinning of the sclera and choroid mostly in the posterior segment, while the sclera in the anterior segment did not differ markedly between myopic eyes and emmetropic eyes [39]. In a similar manner, central corneal thickness was not associated with axial length in previous population studies on adults [40].

Potential limitations of our study should be mentioned. First, we measured only the anterior corneal curvature radius while the posterior corneal curvature radius was not assessed. The study could therefore address only the corneal refractive power anterior of the anterior corneal surface, but not the total refractive power of the cornea. Second, central corneal thickness and anterior chamber depth were not recorded, so that the contribution of these biometric parameters to the optic system of the eyes could not be addressed. Third, our study may not be representative for the whole population of China in view of the heterogeneity of the country.

In conclusion, longer CCR (i.e., flatter corneas) was correlated with male sex, younger age, taller body height, lower paternal educational level, maternal myopia, longer axial length, larger corneas (i.e., longer horizontal corneal diameter), and smaller amount of cylindrical refractive error. These findings may be of interest for elucidation of the process of emmetropization and myopization and for corneal refractive surgery.
